# Screening of Abdominal Aortic Aneurysm Using Portable Transthoracic Echocardiography among Patients with Acute Coronary Syndrome

**DOI:** 10.1155/2020/9510546

**Published:** 2020-06-27

**Authors:** Yousef Shukha, Ofir Koren, Tsafrir Or, Yoav Turgeman, Mahmud Mahamid, Mohamed Jabaren

**Affiliations:** ^1^Internal Medicine Department E, Rambam Medical Center, Haifa, Israel; ^2^Heart Institute, Emek Medical Center, Afula, Israel; ^3^Bruce Rappaport Faculty of Medicine, Technion-Israel Institute of Technology, Haifa, Israel; ^4^Gastrointestinal Diseases Unit, Shaare Zedek Medical Center, Jerusalem, Israel

## Abstract

**Background:**

Abdominal aortic aneurysm (AAA) and acute coronary syndrome (ACS) share common risk factors.

**Objectives:**

To assess the abdominal aortic diameter (AAD) among patients with ACS using transthoracic echocardiography (TTE).

**Methods:**

Patients with ACS admitted to our intensive cardiac care unit from December 2013 to June 2014 were screened prospectively for AAA via AAD measurement in the subcostal TTE view. AAA was defined as an aneurysm with a transverse diameter of ≥30 mm.

**Results:**

Sixty seven patients were included. The male-to-female sex ratio was 7 : 1. The vast majority of patients were admitted due to STEMI (73%), and the rest were equally divided as NSTEMI and unstable angina. The mean patient age was 58.4 ± 10.4 years. AAD measurements were feasible in 57 patients (85%); among them, AAA was diagnosed in six patients (10.5%). The average additional time required to measure the abdominal aorta was 4 ± 1 min. All patients with AAA were men and had a higher prevalence of smoking (83.3% vs. 60.6%, *p* < 0.003) and a lower incidence of diabetes mellitus than those without aneurysm. The prevalence of AAA tended to be related to age (12.5% in those older than 60 years and 18.7% in those older than 65 years).

**Conclusions:**

The overall prevalence of AAA is significantly high among patients with ACS and increases with age. AAA screening as a part of routine cardiac TTE can be easily, rapidly, and feasibly performed and yield accurate findings. AAD measurement in the subcostal view should be implemented as a part of routine TTE in patients with ACS.

## 1. Introduction

Abdominal aortic aneurysm (AAA) is defined as an abnormal vascular dilatation composite of all three endothelial layers of at least 1.5 times the normal diameter or ≥30 mm. The prevalence of AAA worldwide is estimated to be 1%-2% in the general population and increases sharply up to 9% among the elderly population (>65 years) when multiple risk factors are present [1–4].

AAA rupture is the 14th leading cause of death in the United States. Most AAAs carry no signs or symptoms until rupture and are mainly detected with the following signs: pulsating abdominal mass, umbilical murmur on auscultation, or visual findings accidentally obtained via imaging for other reasons [5–8].

AAA rupture usually presents with severe pain and tenderness on palpation; the pain usually radiates to the back, chest, or scrotum, in addition to hypotension and shock [9]. The risk of rupture is related to the size of the aneurysm and the presence of symptoms; it ranges from approximately 0.5%–5% per year for aneurysms <5.0 cm in diameter to 20%–40% per year for those >7 cm in diameter [10–12]. The overall mortality related to AAA rupture is 80%–90% compared with a 30-day postoperative mortality of 3% after elective surgery. Therapeutic options for AAA repair are either by open surgery or endovascular repair (EVAR). Nowadays, EVAR represents the preferable choice for AAA treatment [13].

AAAs are divided according to the origin of renal arteries as suprarenal, juxtarenal, and infrarenal. Approximately, 80% of all AAAs are infrarenal [14]. The main risk factors for AAA are male sex, white race, history of smoking (above 100 cigarettes in a lifetime), and age above 65 years. The other risk factors associated with AAA include a family history of AAA, acute coronary syndrome (ACS), peripheral artery disease, hypercholesterolemia, hypertension, cerebrovascular diseases, and short stature. The factors that reduce the incidence of AAA are female sex, diabetes mellitus, and black race [14].

The most common pathologic process associated with aortic aneurysms is atherosclerosis. At least 90% of all AAAs >4 cm in diameter occur in relation to atherosclerosis, and most of these aneurysms are located below the renal arteries [15, 16].

The current modality of choice for AAA screening is abdominal ultrasound, which has a sensitivity of 95%–100% and specificity of 100% and is a cheap, feasible, accurate, and safe method [16].

Early detection of AAA is related to a 42% reduced mortality according to several studies, and a single lifetime screening of AAA is sufficient if the result is negative [8]. Considering the balance between the incidence of AAA and cost-effectiveness of screening methods, the relatively low prevalence of AAA in the general population (1%-2%) implies targeting of specific high-risk populations [17].

Currently, the indications for AAA screening according to the Society for Vascular Surgery and Society for Vascular Medicine and Biology are male sex and age over 60 years, female sex and age over 60 years with cardiovascular risk factors, and male and female sexes and age over 50 years with a family history of AAA [18].

Transthoracic echocardiography (TTE) is an ultrasound technique that targets the heart and thoracic aorta, with a sensitivity of 91%–96% in detecting AAA using the same probe in the subcostal view [18].

### 1.1. Rationale of the Research

The rationale of this research was based on the fact that ACS and AAA share similar advanced atherosclerotic pathological processes. Our hypothesis was that patients presenting with ACS have significantly higher AAA rates. The objective of this study was to determine the actual prevalence of AAA among patients with ACS and to estimate the utility and feasibility of routine TTE as a screening method for AAA.

## 2. Methods

### 2.1. Research Plan

This cross-sectional study was conducted in the Cardiology Department at the Emek Medical Center. Patient recruitment began in December 2013 and lasted for 7 consecutive months. The study population included patients who were admitted owing to ACS due to ST-segment elevation myocardial infarction (STEMI), non-STEMI, or unstable angina pectoris and scheduled for routine cardiac TTE. Patients were excluded from the study if they were younger than 18 years or pregnant. Echocardiographic technicians were instructed to demonstrate the abdominal aorta, above and below the renal artery origin, in the subcostal view (while the patients were in the supine position) using the same probe and equipment used for cardiac TTE. The abdominal aortic diameter (AAD) was measured using a transverse scale. The measurements were revised by at least two cardiologist experts in cardiac imaging before the final decision was made. AAA was defined as an aneurysm with a transverse aortic diameter of >30 mm. In order to avoid measurement errors, two experienced technicians have been trained by a cardiologist who specializes in echocardiographic imaging.

### 2.2. Recruitment Strategy

Recruitment was performed within 24–48 hours of admission after obtaining informed consent. Eligible patients underwent a personal interview during which they were asked to answer a structured questionnaire that included information on demographics, clinical data, and medical history. Supplemental data were obtained using computerized medical records (“Orion,” “Ofek,” and “Chameleon”).

### 2.3. Echocardiography Technique and Variables

Scanning of the abdominal aorta was performed at the bedside using a portable echocardiography machine (VIVID I General Electrical) with a 2.5 MHz cardiac probe. No specific preparations were needed before the examination. Using the same probe, TTE was performed in the subcostal view to measure the AAD in the transverse plane, while the patients were in the supine position. The diameter was measured as the maximum diameter in the transverse plane in two places: above and below the renal arteries (suprarenal and infrarenal aortic levels, respectively). Three separate measurements for the diameter were taken, and the average measurement was considered.

### 2.4. Statistics

The sample size was assessed considering prior reports of an average prevalence of 6% for AAA (according to the AAD of >30 mm) in high-risk patients. For a minimum of 10% difference between study groups and 80% power for a 95% confidence interval, we needed to include at least 63 patients. The prevalence of AAA was calculated as the number of patients with ACS diagnosed with AAA in relation to the total number of participants in the study. The percentage of patients with successful measurement of the AAD using the TTE probe was used to indicate feasibility. Categorical variables were presented using frequencies and percentages and continuous variables using standardized distribution indices (means, standard deviations, or medians). For comparison of continuous and categorical variables between the study groups, a *t*-test (or Wilcoxon test) and the chi-square test (or Fisher's test) were used, respectively. Statistical processing was performed using the SAS 9.4 software. *p* values of <0.05 were considered significant.

### 2.5. Ethics

The study was approved and monitored by the hospital's ethics committee according to the Helsinki Convention principles (EMC 0152-12). Informed consent was obtained from all patients.

## 3. Results

Sixty seven patients hospitalized owing to ACS in our Cardiac Care Unit from December 2013 to June 2014 underwent TTE for the measurement of the transverse diameter of the abdominal aorta. The average age of the patients was 58.6 ± 10.2 years. Fifty nine patients were men (88%). None of the patients had undergone a previous examination to screening for AAA. AAD measurement by this technique was feasible in only 57 patients (out of the total 67 patients). Among them, AAA (AAD of >30 mm) was diagnosed in 10.5% of patients (6 out of 57 patients). The average additional time required to measure the abdominal aorta was 4 ± 1 min in addition to the standard regular TTE duration. To review the results, we divided the study population into two distinct groups: group 1 (no AAA group) consisting of patients with ACS with a normal AAD and group 2 (AAA group) consisting of patients with ACS with newly diagnosed AAA.

All patients with AAA were men (100% vs. 83.3%, *p* < 0.47). The overall median patient age did not differ between the groups; however, the prevalence of AAA seemed to be related to age. Of the six patients with AAA, five (83%) were older than 50 years. Three patients were older than 60 years, and three patients were older than 65 years (Table 1).

More than 80% of the patients with AAA presented with STEMI, and the remaining patients (16.6%) had unstable angina (83.3% vs. 63.9%, *p* < 0.003).

Among the known atherosclerotic risk factors, smoking appeared to be a significant risk factor for AAA (83% vs. 60.6%, *p*=0.03). The shared risk factors of AAA and ACS were obesity, hypertension, hypercholesterolemia, family history of coronary artery disease, and peripheral vascular disease. Conversely, the incidence of diabetes mellitus was lower in group 2 but without a significant difference (33.3% vs. 40.9%, *p* = NS).

Group 2 had a significantly higher rate of prior coronary artery bypass graft surgery (33.3% vs. 4.5%, *p* < 0.004) and a nonsignificantly higher rate of prior myocardial infarction or cerebrovascular accident than group 1 (33.3% vs. 4.9% and 16.6% vs. 9.8%, respectively, *p* = NS for both).

The degree of coronary artery disease involvement and echocardiographic parameters was not found to be related to the appearance of AAA. Group 2 had a lower average ejection fraction (40% ± 10% vs. 50% ± 10%) and higher left ventricular end-diastolic (48.3 ± 2.3 vs. 32.2 ± 5.9 mm) and systolic diameters (33.4 ± 2.6 vs. 32.2 ± 5.9 mm) than group 1; however, all were found to have no significant difference.

In group 2, the average AAD was 29.3 ± 3.5 mm above the renal artery (two out of six patients) and 31.3 ± 4.4 mm below the renal artery (six out of six patients) in comparison with the average AAD of 22.6 ± 3.1 mm above the renal artery and 23.3 ± 3 mm below the renal artery in group 1 (*p*=0.003).

## 4. Discussion

ACS patients are at high risk of developing atherosclerotic complications, and among them, and perhaps, the most dangerous of all is AAA. Since AAA is a process that is mostly asymptomatic until rupture occurs, early screening is essential to minimize the significantly high mortality rates.

Our study aimed to measure the feasibility and efficiency of TTE in detecting AAA among ACS patients and examine the overall prevalence in this subgroup. Furthermore, by gathering the medical history and characteristics of the patients with ACS, we aimed to identify an independent risk factor for AAA development, which will be used as a screening tool.

The present study was based on the fundamental similarity between TTE and abdominal ultrasound, which is currently the gold standard modality for AAA screening; we used regular TTE instead of abdominal ultrasound, in the same position using the same probe, to screen for AAA.

Extending the use of routine TTE for patients with ACS to assess the abdominal aorta in the subcostal view was convenient and easy to perform. No specific preparations from the patients or the examiner, additional costs (same equipment), or additional personnel (same examiner) were needed. Furthermore, it required only an additional time of 4 ± 1 min in addition to the standard TTE duration. The feasibility of the technique was high in 85% of the patients. Most of the failed imaging attempts resulted from a poor acoustic window and low echogenicity.

**Figure 1:**
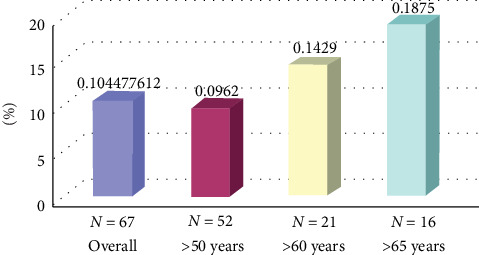
Prevalence of abdominal aortic aneurysm in the overall population in relation to age.

This modality proved its usefulness and efficiency by detecting asymptomatic AAA in more than 10% of the patients in the study group. Although the study population was not large, the prevalence among the patients with ACS was much higher than the known prevalence (Figure 1).

Our findings were similar to that of the study by Caroline Cueff [8] that used this technique to screen for AAA among ACS patients and found out that it only required additional 2-3 minutes in average in addition to the standard TTE duration, and no extra personnel or cost were needed.

Giaconi et al. [16] found a feasibility rate (93%) higher than that in our study, which can be explained by the inclusion of multiple echocardiography personnel in the study and their differences in seniority and experience.

### 4.1. Study Limitations

A major limitation of our study was the small sample size, which, in our opinion, had a major influence on the statistical significance of the minor endpoints, i.e., patients' risk factors and echocardiographic parameters. Moreover, our study would have gained more power if we added a comparison with a clinically matched group without any history of ACS.

## 5. Conclusions

The prevalence of AAA among the patients admitted to our intensive cardiac care unit was found to be twice higher than that among the age-matched patients. It was also found to be dependent on age, and the elderly patients (>65 years) seemed to be at a particularly high risk. The patients admitted owing to STEMI who were smoking and underwent prior cardiac surgery had a higher incidence of AAA.

TTE as a screening method for AAA in patients with ACS as a part of routine cardiac examination can be easily, rapidly, and feasibly performed and yield accurate findings. AAD measurement in the subcostal view should be implemented as a part of routine TTE in patients with ACS.

## Figures and Tables

**Table 1:** Baseline patient characteristics.

Patient characteristics	Group 1 (no AAA), *N* = 61 (%)	Group 2 (AAA), *N* = 6 (%)	*p* value
Age (year)	58.4 ± 10.4	59 ± 10	NS
Male sex	53 (86)	6 (100)	NS

*Risk factors*			
Smoking	37 (60)	5 (83)	0.0371
Hypertension	35 (57)	4 (66)	NS
Hypercholesterolemia	43 (70)	5 (83)	NS
Diabetes mellitus	25 (40)	2 (33)	NS
Family history of coronary artery disease	26 (42)	4 (66.6)	NS
Body mass index	27.6 ± 3.8	28 ± 2.6	NS
PVDx	5 (8.1)	1 (16)	NS

*Medical history*			
Coronary artery disease	22 (36)	3 (50)	NS
Myocardial infarction	15 (24)	2 (33)	NS
Coronary artery bypass graft	3 (4)	2 (33)	<0.004
Cerebrovascular accident	6 (9)	1 (16)	NS

*Type of acute coronary syndrome*			
STEMI	39 (63)	5 (83)	NS
Non-STEMI	20 (32)	0 (0)	NS
Unstable angina	2 (3)	1 (16)	NS

*Extent of coronary artery disease*			
One-vessel disease	10 (16)	1 (16)	NS
Two-vessel disease	4 (6)	0 (0)	NS
Three-vessel disease	5 (8)	2 (33)	NS
No angiography	42 (68)	3 (50)	NS

*Echocardiographic findings*			
Ejection fraction (%)	50 ± 10	40 ± 10	NS
Intraventricular septum (mm)	10.9 ± 1.3	11.1 ± 1.3	NS
Posterior wall width C (mm)	10.1 ± 1.2	9.7 ± 1.3	NS
Left ventricular end-diastolic diameter (mm)	46.9 ± 4.8	48.3 ± 2.3	NS
Left ventricular end-systolic diameter (mm)	32.3 ± 5.9	33.4 ± 2.6	NS
Left atrial diameter (mm)	37.3 ± 4	38 ± 2.4	NS

*Abdominal aortic diameter*			
Suprarenal	22.6 ± 3.1	29.3 ± 3.5	0.0003
Infrarenal	23.3 ± 3	31.3 ± 3.8	0.0003

AAA: abdominal aortic aneurysm; STEMI: ST-segment elevation myocardial infarction; PVD: peripheral artery disease.

## Data Availability

The data that support the findings of this study are available from the corresponding author, Y. S, upon reasonable request.

## References

[1] Bergqvist D., Björck M., Wanhainen A. (2008). Abdominal aortic aneurysm—to screen or not to screen. *European Journal of Vascular and Endovascular Surgery*.

[2] Johnston K. W., Rutherford R. B., Tilson M. D., Shah D. M., Hollier L., Stanley J. C. (1991). Suggested standards for reporting on arterial aneurysms. *Journal of Vascular Surgery*.

[3] Gillum R. F. (1995). Epidemiology of aortic aneurysm in the United States. *Journal of Clinical Epidemiology*.

[4] Ouriel K., Green R. M., Donayre C., Shortell C. K., Elliott J., DeWeese J. A. (1992). An evaluation of new methods of expressing aortic aneurysm size: relationship to rupture. *Journal of Vascular Surgery*.

[5] Lindholt J. S., Norman P. (2008). Screening for abdominal aortic aneurysm reduces overall mortality in men. A meta-analysis of the mid- and long-term effects of screening for abdominal aortic aneurysms. *European Journal of Vascular and Endovascular Surgery*.

[6] Thorbjørnsen K., Svensjö S., Djavani Gidlund K., Gilgen N., Wanhainen A. (2019). Prevalence and natural history of and risk factors for subaneurysmal aorta among 65-year-old men. *Upsala Journal of Medical Sciences*.

[7] Villard C., Hultgren R. (2018). Abdominal aortic aneurysm: sex differences. *Maturitas*.

[8] Cueff C., Keenan N. G., Krapf L. (2011). Screening for abdominal aortic aneurysm in coronary care unit patients with acute myocardial infarction using portable transthoracic echocardiography. *European Heart Journal-Cardiovascular Imaging*.

[9] Roshanali F., Mandegar M. H., Yousefnia M. A., Mohammadi A., Baharvand B. (2007). Abdominal aorta screening during transthoracic echocardiography. *Echocardiography*.

[10] Brewster D. C., Cronenwett J. L., Hallett J. W., Johnston K. W., Krupski W. C., Matsumura J. S. (2003). Guidelines for the treatment of abdominal aortic aneurysms: report of a subcommittee of the joint council of the American association for vascular surgery and society for vascular surgery. *Journal of Vascular Surgery*.

[11] Sprynger M., Willems M., Van Damme H., Drieghe B., Wautrecht J. C., Moonen M. (2019). Screening program of abdominal aortic aneurysm. *Angiology*.

[12] Aggarwal S., Qamar A., Sharma V., Sharma A. (2011). Abdominal aortic aneurysm: a comprehensive review. *Experimental and Clinical Cardiology*.

[13] Kobzantsev Z., Bass A. (2019). Laparoscopic aortic surgery. *The Israel Medical Association Journal*.

[14] Chiesa R., Maria Marone E., Brioschi C., Frigerio S., Tshomba Y., Melissano G. (2006). Open repair of pararenal aortic aneurysms: operative management, early results, and risk factor analysis. *Annals of Vascular Surgery*.

[15] Navas E. V. (2012). Abdominal aortic aneurysm screening during transthoracic echocardiography: cardiologist and vascular medicine specialist interpretation. *World Journal of Cardiology*.

[16] Giaconi S., Lattanzi F., Orsini E., Prosperi R., Tartarini G. (2003). Feasibility and accuracy of rapid evaluation of the abdominal aorta during routine transthoracic echocardiography. *Italian Heart Journal Supplement*.

[17] Dupont A., Elkalioubie A., Juthier F. (2010). Frequency of abdominal aortic aneurysm in patients undergoing coronary artery bypass grafting. *The American Journal of Cardiology*.

[18] Bambgartner I., Hirsch A. T., Abola M. T. (2008). Cardiovascular risk profile and outcome of patients with an abdominal aortic aneurysm in out-patients with atherothrombosis: data from the reduction of atherothrombosis for continued health (REACH) registry. *Journal of Vascular Surgery*.

